# Associated-risk determinants for anthroponotic cutaneous leishmaniasis treated with meglumine antimoniate: A cohort study in Iran

**DOI:** 10.1371/journal.pntd.0007423

**Published:** 2019-06-12

**Authors:** Mohammad Reza Aflatoonian, Iraj Sharifi, Behnaz Aflatoonian, Mehdi Bamorovat, Amireh Heshmatkhah, Zahra Babaei, Pooya Ghasemi Nejad Almani, Mohammad Ali Mohammadi, Ehsan Salarkia, Abbas Aghaei Afshar, Hamid Sharifi, Fatemeh Sharifi, Ahmad Khosravi, Mehrdad Khatami, Nasir Arefinia, Alireza Fekri, Saeideh Farajzadeh, Ali Khamesipour, Mehdi Mohebali, Mohammad Mehdi Gouya, Mohammad Reza Shirzadi, Rajender S. Varma

**Affiliations:** 1 Research Center of Tropical and Infectious Diseases, Kerman University of Medical Sciences, Kerman, Iran; 2 Leishmaniasis Research Center, Kerman University of Medical Sciences, Kerman, Iran; 3 Shahid Dadbin Clinic, Kerman University of Medical Sciences, Kerman, Iran; 4 Research Center for Hydatid Disease in Iran, Kerman University of Medical Sciences٫ Kerman, Iran; 5 HIV/STI Surveillance Research Center, and WHO Collaborating Center for HIV Surveillance, Institute for Futures Studies in Health, Kerman University of Medical Sciences, Kerman, Iran; 6 Pharmaceutics Research Center, Institute of Neuropharmacology, Kerman University of Medical Sciences, Kerman, Iran; 7 School of Medicine, Bam University of Medical Sciences, Bam, Iran; 8 Department of Dermatology, Afzalipour Hospital, Leishmaniasis Research Center, Kerman University of Medical Sciences, Kerman, Iran; 9 Center for Research and Training in Skin Diseases and Leprosy, Tehran University of Medical Sciences, Tehran, Iran; 10 Department of Medical Parasitology and Mycology, School of Public Health, Tehran University of Medical Sciences, Tehran, Iran; 11 Center of Disease Control (CDC), Ministry of Health, Tehran, Iran; 12 Regional Centre of Advanced Technologies and Materials, Faculty of Science, Palacký University in Olomouc, Šlechtitelů 27, Czech Republic; Insitut Pasteur de Tunis, TUNISIA

## Abstract

**Background:**

The control of cutaneous leishmaniasis (CL) is facilitated by knowledge of factors associated with the treatment failures in endemic countries. The aim of this evaluation was to identify the potential risk determinants which might affect the significance of demographic and clinical characteristics for the patients with anthroponotic CL (ACL) and the outcome of meglumine antimoniate (MA) (Glucantime) treatment.

**Methodology/Principal findings:**

This current was executed as a cohort spanning over a period of 5 years which centered in southeastern part of Iran. Altogether, 2,422 participants were evaluated and 1,391 eligible volunteer patients with ACL caused by *Leishmania tropica* were included. Overall, 1,116 (80.2%) patients received MA intraleisionally (IL), once a week for 12 weeks along with biweekly cryotherapy, while 275 (19.8%) patients received MA alone (20 mg/kg/day for 3 weeks) (intramuscular, IM). The treatment failure rate in ACL patients was 11% using IL combined with cryotherapy plus IM alone, whilst 9% and 18.5% by IL along with cryotherapy or IM alone, respectively. Multivariate logistic regression model predicted 5 major associated-risk determinants including male (odds ratio (OR) = 1.54, confidence interval (CI) = 1.079–2.22, p = 0.018), lesion on face (OR = 1.574, CI = 1.075–2.303, p = 0.02), multiple lesions (OR = 1.446, CI = 1.008–2.075, p = 0.045), poor treatment adherence (OR = 2.041, CI = 1.204–3.46, p = 0.008) and disease duration > 4 months (OR = 2.739, CI = 1.906–3.936, p≤0.001).

**Conclusions/Significance:**

The present study is the original and largest cohort of ACL patients who treated with MA. A comprehensive intervention and coordinated action by the health authorities and policy-makers are crucial to make sure that patients strictly follow medical instructions. Early detection and effective therapy < 4 months following the onset of the lesion is critical for successful treatment of the patients. Since a significant number of patients are still refractory to MA, reducing man-vector exposure and development of new effective alternative drugs are essential measures against ACL due to *L*. *tropica*.

## Author summary

Cutaneous leishmaniasis (CL) is a serious neglected tropical disease with social stigma and associated disfiguring with health burden, especially in poor endemic regions of the world. Iran is among the seven high burden CL-infected countries. Limited data are available in regarding to CL treatment and related risk determinants. Parasitologically confirmed ACL patients caused by *Leishmania tropica* were treated over a 5-year period (2012–2016), with IL meglumine antimoniate (MA) (Glucantime), combined with cryotherapy or with IM MA alone. The objective of this study was to identify the potential risk factors that are associated with response to treatment. The ensuing results with both therapeutic routes identified 5 major risk determinants namely male patients, lesion on face, multiple lesions, poor treatment regimen and disease duration >4 months. The extent to which medical instructions related to patients, therapy and the healthcare system should be seriously monitored. This requires multidisciplinary actions to address specific barriers which directly threaten the treatment outcome. Furthermore, early detection and prompt treatment <4 months following the disease duration together with implementations of public health education and prophylactic measures should receive priority in high risk areas.

## Introduction

Leishmaniasis is a neglected disease with growing social and public health concern [1] in many parts of the tropical and subtropical countries, especially in the Eastern Mediterranean Basin such as Iran. Cutaneous leishmaniasis (CL) is the most prevalent form of the disease which comprises approximately 70–75% of the total global cases [2]. Recent studies demonstrated that CL increased significantly and reached hyper-endemic levels in the Middle East war zones of Afghanistan, Pakistan, Iraq and Syria [3] and concurrently affecting refugees from those areas [4]. It is suggested that if the scarred CL cases are considered in the prevalence estimates of global burden of disease reports (nearly 40 million cases), instead of those simply with active infection (approximately 4 million patients) [1,5], then the overall burden of the disease as measured by the Disability Adjusted Life Years (DALY) would be increased by a factor of 10. Two species are mainly responsible for CL in Iran: anthroponotic CL (ACL) caused by *Leishmania tropica*, transmitted by *Phlebotomus sergenti* and zoonotic CL (ZCL) which is caused by *Leishmania major* and transmitted by *Ph*. *papatasi* and small rodents are the main reservoirs primarily in rural life cycle [6]. Currently, there is no vaccine available against any form of leishmaniasis. Control of CL is complex due to the diversity of *Leishmania* species, biological vectors and reservoir hosts. Various risk determinants play crucial roles in the proliferation of the disease both in respect of increasing incidence rate and spreading of the disease to new foci. Such risk factors consist of environmental modification, host immune status, travel/migration, population displacement, drug resistance and parasite species [5,7–9].

Standard treatment of CL is pentavalent antimonial (SbV) which is not always effective and resistance have been reported; this is mainly attributed to parasite species, host factors and quality and quantity of the treatment measures [8,10–12]. Control of ACL caused by *L*. *tropica* is primarily based on early detection of the cases, diagnosis, identification of the causative agent, and prompt treatment of the patients via an effective surveillance system. Given that humans are the only reservoir host, untreated chronic cases such as leishmaniasis recidivans (lupoid leishmaniasis), remain the infective reservoir for the dissemination of the organism [13,14].

Over the last decades, chemotherapy using antimonials remains the only and first-choice management approach for various forms of CL. Unfortunately, the compliance of the basic recommended treatment is low, mainly due to the long duration of therapy, toxic effects, painful parenteral administration, poor therapeutic response and the emergence of resistance [10,14,15]. Cutaneous leishmaniasis response to meglumine antimoniate (MA) (Glucantime) is rather poor in Iran, treatment failures and relapses [10,16,17] have been documented following MA treatment from various endemic regions. Public health surveillance personnel could be an essential tool for rigorous case-detection approaches to address critical concerns.

This study was aimed to evaluate the associated-risk determinants which might potentially affect the treatment outcome in a major ACL focus in southeastern Iran. We conducted this prospective cohort study reporting on treatment outcomes of ACL due to *L*. *tropica* to identify the potentially effective treatment regimen.

## Methods

### Ethical considerations

The project was reviewed and approved by the Ethics Committees of the Leishmaniasis Research Center and the Institutional Review Board (IRB) of Kerman University of Medical Sciences; protocol no. 94/305 and Ethics no. IR. KMU. REC. 1394. 276, respectively. A national guideline for management of leishmaniasis was developed based on WHO recommendations. The patients with proven parasitological CL were treated according to the national leishmaniasis guideline free of charge. A short prophylactic and therapeutic educational training program was planned during the first visit of the patients by face-to-face communications to familiarize the patient with the clinical characteristics of the disease, risk-associated factors, treatment approaches, the probable relapses and other possible control measures related to CL. The patients were advised to stick to the follow up schedule and final assessment. Patients, who were suspected of having other diseases, were referred to the Kerman University of Medical Sciences Hospitals for further diagnosis and proper treatment. The patients only received routine diagnosis and treatment free of charge and no extra intervention was performed. The reasons to obtain oral instead of written consent was that the residents were mostly illiterate; moreover, the patients only received routine treatment and if we asked them to sign written consent some of the patients might refuse to participate and receive treatment, although they orally accepted to participate. Since the disease is anthroponotic, the Ministry of Health (MoH) emphasizes to treat all patients to prevent further dissemination of ACL. A case report form (CRF) was completed for every patient; demographic characteristics including sex, age, nationality and also clinical data such as location, number and dressing of the lesion(s), route and duration of treatment and the outcome of treatment failure and relapse, were recorded. In the CRF, the patient was asked if she/he permits publication of her/his disease information including lesion photo(s).

This large scale project was carried out due to recent consecutive earthquakes in southeastern Iran with consequential environmental changes and population displacement.

### Design and patient recruitments

This study was conducted as a cohort study from March 2012 to January 2016 at Dadbin Health Clinic, affiliated with the School of Medicine, Kerman University of Medical Sciences and Kerman Leishmaniasis Research Center, in southeastern Iran.

**Fig 1:**
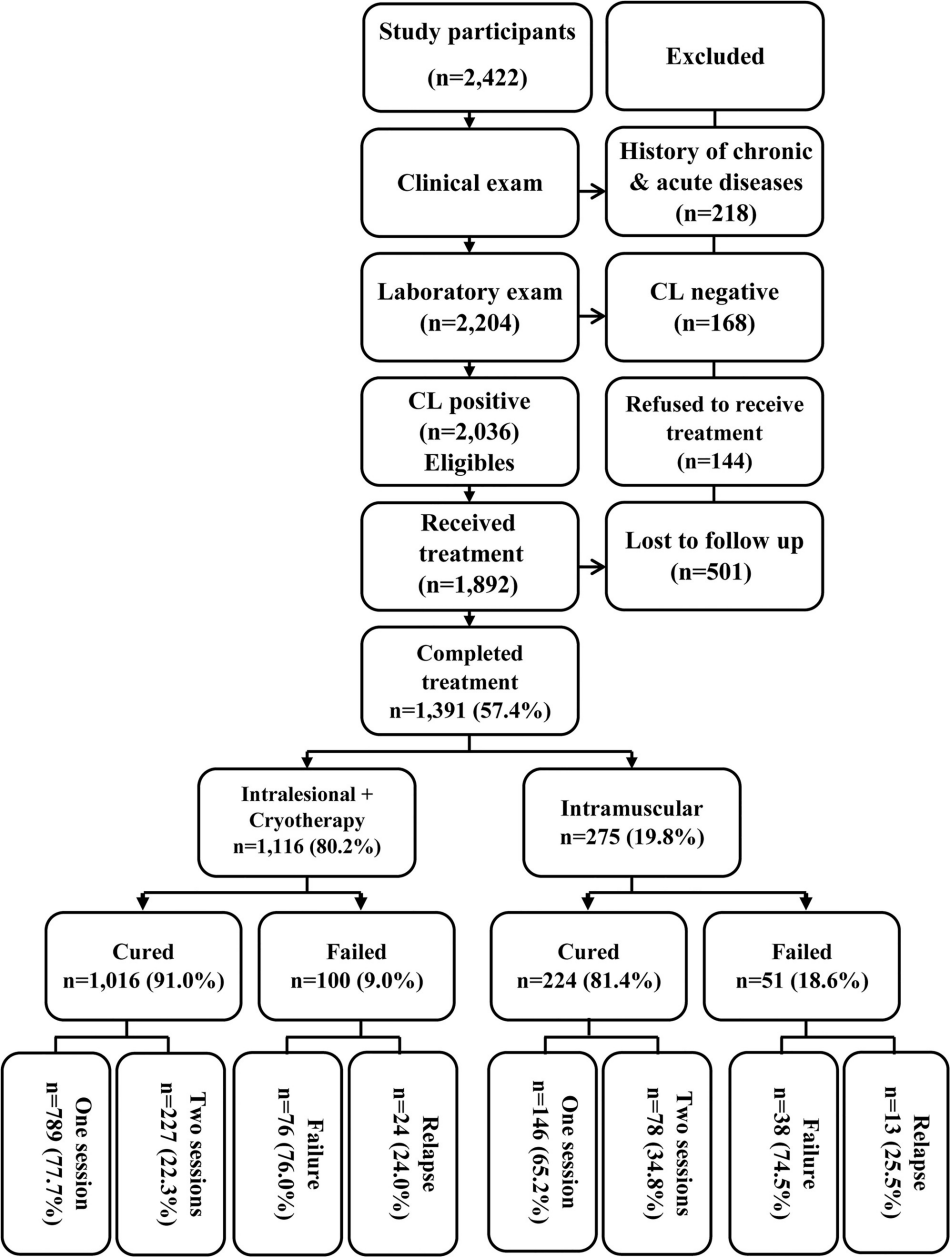
Screening profile and treatment outcome.

The clinic manages CL patients who referred from different endemic areas of the province. Only patients with the first episode of CL were included to avoid various biases and confounders linked to clinical failure. Male and female patients with all age groups were recruited. The screening profile and treatment outcome is summarized in Fig 1. Totally, 2,422 participants were screened, interviewed and physically examined. At this stage, 218 patients (9%) with a history of chronic or acute diseases were excluded and 2,204 patients with suspected CL lesion were referred to the diagnostic laboratory for confirmation. Overall, lesion of 168 (6.9%) of the patients was not CL while lesions of 2,036 of the patients (84.1%) showed a history of CL.

Initially, 144 (5.9%) of the patients refused to receive treatment, of the remaining (n = 1,892), 501 subjects (26.5%) were lost to follow up for the following reasons: migration, death, absence and withdrawal consent n = 231; allergic reaction, acute and underlying chronic diseases, n = 69; non-residents patients from other districts and provinces, n = 119 and those who received other treatment modalities, n = 82. Overall, a total of 1,391 (57.4%) eligible patients completed the study (S1 Checklist).

### Confirmation of CL cases

Skin scraping tissues were collected from the periphery of an active lesion and two glass slide smears were prepared; one smear was fixed with methanol and stained by Giemsa for direct microscopic examination and the other smear was used for identification of the causative agent using nested-PCR.

### Molecular identification

From a total of 1,391 positive smear preparation samples, 207 slide smear preparations were randomly selected for DNA extraction using the high pure Template Purification Kit (Roche, Germany) and identification of *Leishmania* parasite using PCR methods.

The nested–PCR assay was basically performed according to the previously described method [18]. Briefly, two consecutive sets of general and specific primers were used for amplification of variable mini circle kinetoplast DNA fragments (kDNA). Products were visualized from the second round of PCR on 1.5% gel electrophoresis.

### Treatment regimen

The treatment of parasitological proven cases was done by daily administration of 20 mg/kg of MA, at most 3 ampoules per day IM for 21 days. Some of the patients received IL administration of MA into the base of the lesion by a fine gauge needle (25G), every week for a maximum of 12 weeks along with cryotherapy using liquid nitrogen which was given biweekly for a maximum of 12 weeks [6]. The patients who received IL treatment were actively referred to the designated health centers for injection, and if they did not come to the centers for any reason, the personnel of these centers called them or visited at home. Whilst, in case of systemic injections the ampoules were delivered to the patients to receive the medication in the nearest rural or urban health clinic, although the patients had to deliver their checklists and empty ampoules to the responsible heath personnel indicating the date and the number of injections. The treatment strategy was based on national guideline which was originated from WHO protocol with minor modifications [14]. The criteria for assigning IM route was the number of lesions > 5 and/or lesions ≥ 3 cm in diameter or the lesion close to vital organs.

### Treatment failure and cure

The criteria for cure were complete re-epithelialization of every lesion (nodule, plaque or ulceration) following therapy with one or two courses of MA at three months post-treatment follow–up assessment. Treatment failure was defined as patients who demonstrated active lesion(s) or relapse at three months follow-up visit.

### Relapse

Reappearance of a nodule, plaque or ulceration following cure.

### Poor treatment adherence

Poor treatment adherence was referred to the patients who did not follow the instruction of treatment recommendation or received the medication partially and not according to the national guideline, or the patients who missed the scheduled appointments; although, both groups received two-thirds of the treatment schedules.

### Statistical analysis

The data was analyzed using a SPSS software version 21 (Chicago, IL, USA) and standard statistical tests were used to assess the significance of the differences between proportion (χ^2^) and means (t-test). Overall, 10 potential demographic and clinical risk determinants for patients with ACL and MA (intralesional and intramuscular collectively or alone) treatment outcome were evaluated. The incidence of MA failure among sexes, age groups, nationality, patients with lesion on the face *vs*. cases with lesion on other locations, multiple lesions *vs*. single lesion, lesions' size, lesion dressing, complete treatment adherence *vs*. poor treatment adherence, duration of lesion(s) and patients with IL *vs*. IM injections. P<0.05 was considered as statistically significance. Odd ratio (OR) is the ratio of treatment outcome in the patients who received IM MA *vs*. IL MA injection plus cryotherapy.

The univariate logistic regression method was used to analyze variables individually and the probability of variables to be used in multivariate logistic regression. Therefore, variables with p- value <0.2 were analyzed by the multivariate for controlling confounding variables and assessment of any possible association. Then, the backward elimination stepwise process was used to obtain the better possible model. Kaplan-Meier curve was used to present survival experience of two treatments (IL and IM) over time. Log-rank test was applied to compare survival curves. Analysis of Kaplan-Meier graphical presentation and log-rank test was performed using Stata 14.2.

## Results

Over the 5-year period (March 2012 to July 2016), 2,422 potential candidates who agreed to participate in the study were interviewed and physically examined for the presence of scar resembling CL, history of an active lesion and any health problem. The overall population of the county was 724,058 most of the subjects were younger than 25 years old (56%) and the minority was ≥ 65 years old (4.5%). Nonetheless, there was no significant difference among various age groups of the participants. A total of 1,892 CL patients were enrolled to receive the treatment modalities. Overall, 501 of the patients were excluded due to various aforementioned reasons. Altogether, over the 5-year period, 1,391 (57.4%) patients completed the treatment; 1,116 (80.2%) received IL injections plus cryotherapy and the remaining, 275 (19.8%) received MA alone intramuscularly (Fig 1).

### Demographical characteristics

Eligible patients comprised 701 males (50.4%) and 690 females (49.6%). The proportion of male to female population was similar and the majority of the patients were Iranian (n = 1,245, 89.5%), whereas the remaining (n = 146, 10.5%) were Afghan migrants (Table 1) (S1 Table). There was no significant difference between the demographic characteristics and ACL disease. The mean age of 1,391 patients was 25.55±19.35 years old, equally distributed among those who responded to the treatment (mean age: 25.72±19.16 years) and those who failed to cure (mean age: 24.12±20.82 years).

**Table 1:** Baseline characteristics of eligible patients and the clinical status of patients with anthroponotic cutaneous leishmaniasis.

Variable	Total Treated patients No (%)	Excluded patients No (%)	Included patients No (%)
**Sex**			
Male	957 (50.6)	256 (51.1)	701 (50.4)
Female	935 (49.4)	245 (48.9)	690 (49.6)
**Age**			
< 25	1,060 (56)	294 (58.7)	766 (55.1)
≥ 25	832 (44)	207 (41.3)	625 (44.9)
**Nationality**			
Iranian	1,665 (88)	420 (83.8)	1,245 (89.5)
Afghani	227 (12)	81 (16.2)	146 (10.5)
**Location**			
Face	481 (25.4)	124 (24.8)	357 (25.7)
Others	1,411 (74.6)	377 (75.2)	1,034 (74.3)
**Number of lesion**s			
1	1,212 (64.1)	322 (64.3)	890 (64)
≥ 2	680 (35.9)	179 (35.7)	501 (36)
**Size of lesion (mm)**			
≤ 10	1,146 (60.6)	339 (67.7)	807 (58)
> 10	746 (39.4)	162 (32.3)	584 (42)
**Total**	1,892 (100)	501 (100)	1,391 (100)

### Clinical status

**Fig 2:**
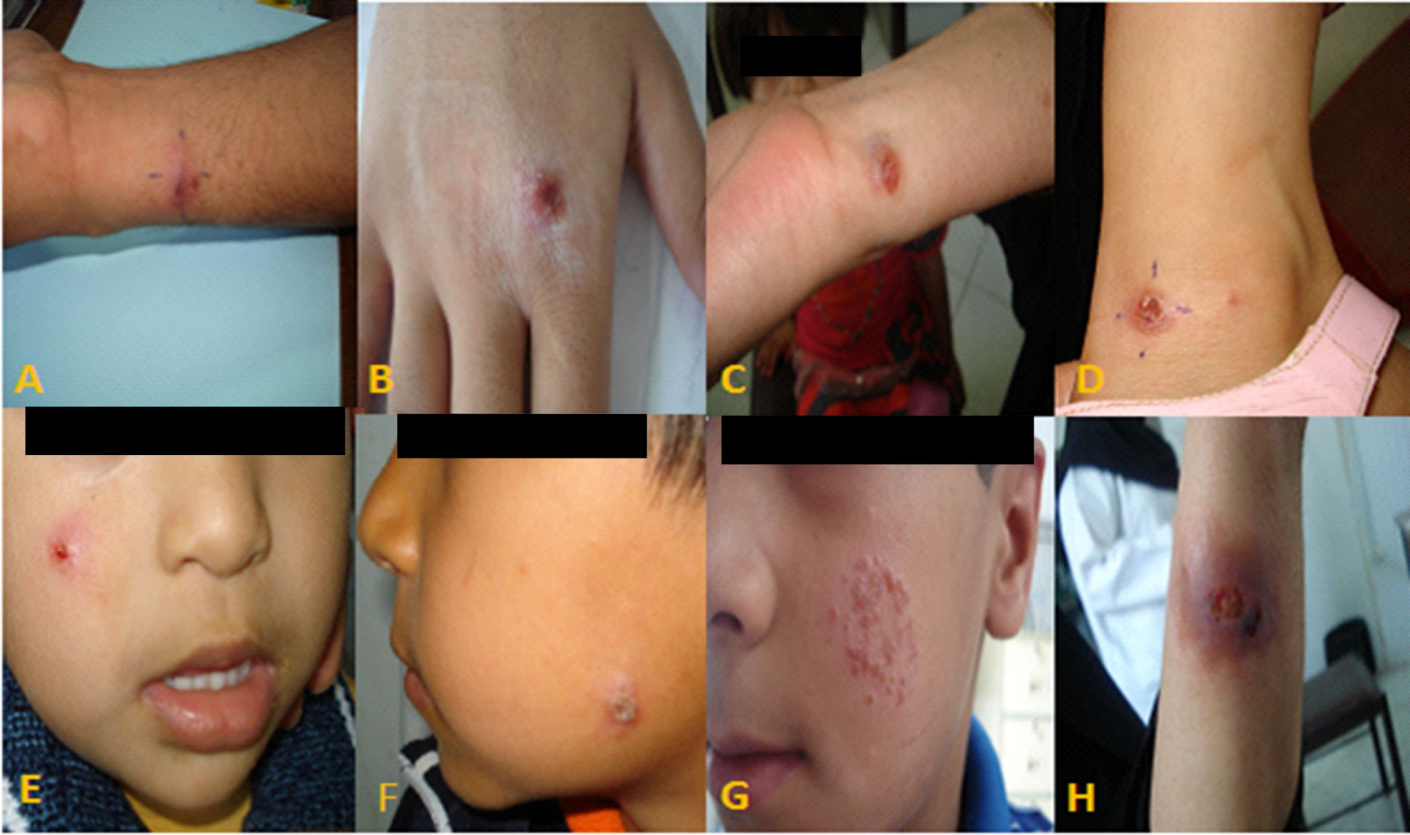
Representative images of patients with anthroponotic cutaneous leishmaniasis in endemic areas from southeastern Iran (A-F; localized cutaneous leishmaniasis lesions, G; leishmaniasis recidivans (lupoid leishmaniasis), H; a non-healing skin lesion.

Clinical characteristics of the patients are presented in Table 1. Most of the lesions were on the hands (47.5%), followed by the face (25.4%), legs (9.7%) and other parts of the body (17.4%) (Fig 2). The majority of ACL patients showed single lesion (64.0%), 19.5% showed two lesions and the rest of the patients showed ≥ 3 lesions (16.5%). The mean size of the lesions (induration or ulcer) was 14.4 mm and most of the lesions were less than 10 mm (58.0%) in diameter and the remaining (42%), > 10 mm. In general, there was no significant difference between the clinical status and the disease.

### Treatment Outcome

Overall, n = 1,116 (80.2%) and n = 275 (19.8%) received intralesional MA plus cryotherapy and intramuscular MA treatment, respectively. Of those who received the treatment protocol by IL route plus cryotherapy, 91% showed cure, either by one course (77.7%) or two courses (22.3%) of treatment. In contrast, the cure rate in patients who received systemic MA alone was 81.4% including 65.2% and 34.8% with one or two courses of treatment which was significantly (p < 0.05) lesser than those with IL route. A similar proportion of relapse was observed among the two treatment regimens (IL 24.0% *vs*. IM 25.5%).

### Risk determinants

Total of 1,391 ACL patients 1,116 patients who received intralesional MA along with cryotherapy, and 275 patients who received intramuscular MA alone, were assessed for potential demographic and clinical treatment risk determinants (Table 2).

**Table 2:** Demographic and clinical characteristics of patients with anthroponotic cutaneous leishmaniasis receiving intramuscular and intralesional meglumine antimoniate, treatment outcome associated risks.

Variable	Treated No (%)	Cure No (%)	Failure No (%)	Univar ate OR(95%CI)	P-value	Multivariate OR(95%CI)	P-value
**Sex**							
Male	701	609(86.9)	92(13.1)	1.616(1.144–2.282)	0.006	1.548(1.079–2.22)	0.018
Female	690	631(91.4)	59(8.6)	1		1	
**Age**							
>25	766	670(87.5)	96(12.5)	1.485(1.047–2.107)	0.027	1.383(0.954–2.004)	0.087
≥25	625	570(91.2)	55(8.8)	1		1	
**Nationality**							
Iranian	1245	1106(88.8)	139(11.2)	1.403(0.758–2.599)	0.281		
Afghani	146	134(91.8)	12(8.2)	1			
**Location**							
Face	357	305(85.4)	52(14.6)	1.61(1.124–2.307)	0.009	1.574(1.075–2.303)	0.02
Others	1034	935(90.4)	99(9.6)	1		1	
**Number of lesion**s							
1	890	801(90)	89(10)	1		1	
≥2	501	439(87.6)	62(12.4)	1.271(0.901–1.794)	0.172	1.446(1.008–2.075)	0.045
**Size of lesion (mm)**							
≤10	807	718(89)	89(11)	1.044(0.74–1.471)	0.807		
>10	584	522(89.4)	62(10.6)	1			
**Dressing of the lesion**							
Yes	1077	962(89.3)	115(10.7)	1			
No	314	278(88.5)	36(11.5)	1.083(0.728–1.612)	0.693		
**Treatment regimen**							
Complete treatment adherence	1291	1162(90)	129(10)	1		1	
Poor treatment adherence	100	78(78)	22(22)	2.541(1.53–4.218)	≤ 0.001	2.041(1.204–3.46)	0.008
**Duration of lesion** **(month)**							
≤4	770	719(93.4)	51(6.6)	1		1	
>4	621	521(83.9)	100(16.1)	2.706(1.896–3.862)	≤ 0.001	2.739(1.906–3.936)	≤0.001
**Treatment route**							
Intramuscular	275	224(81.5)	51(18.5)	2.313(1.603–3.339)	≤ 0.001		
Intralesional	1116	1016(91)	100(9)	1			

### Treatment with combined intralesional and intramuscular MA

The univariate analysis showed 6 major risk determinants including male (OR = 1.616, CI = 1.144–2.282, p = 0.006), age < 25 (OR = 1.485, CI = 1.047–2.107, p = 0.027), face (OR = 1.61, CI = 1.124–2.307, p = 0.009), patients with poor treatment regimen (OR = 2.541, CI = 1.53–4.218, p≤0.001), patient with disease duration, > 4 months seeking treatment modality (OR = 2.706, CI = 1.896–3.862, p≤0.001) and intramuscular route (OR = 2.313, CI = 1.603–3.339, p≤0.001), were significantly associated with the treatment failure (Table 2).

The multivariate regression analysis model confirmed only 5 major risk determinants including males (OR = 1.548, CI = 1.079–2.22, p = 0.018) than females, lesion on face (OR = 1.574, CI = 1.075–2.303, p = 0.02) than other anatomical locations, multiple lesions (OR = 1.446, CI = 1.008–2.075, p = 0.045) than single lesion, poor treatment adherence (OR = 2.041, CI = 1.204–3.46, p = 0.008) than complete treatment regimen and duration of lesion in the patients referred > 4 months (OR = 2.739, CI = 1.906–3.936, p≤0.001) than lesion with ≤ 4 months of age, were significantly associated with treatment failure for ACL patients who treated collectively with intralesional administration coupled with cryotherapy plus meglumine antimoniate alone.

### Intralesional plus cryotherapy administration

Among the patients with ACL (n = 1,116), multivariate regression models showed 4 similar risk- determinants: male (OR = 1.708, CI = 1.108–2.632, p = 0.015), age <25 years old (OR = 1.865, CI = 1.182–2.943, p = 0.007), poor treatment adherence (OR = 3.016, CI = 1.22–7.447, p = 0.017) and duration of lesion > 4 months (OR = 2.679, CI = 1.745–4.112, p≤ 0.001), were significantly associated with the treatment failure (Table 3).

**Table 3:** Associated-risk determinants for anthroponotic cutaneous leishmaniasis patients treated with intralesional meglumine antimoniate along with cryotherapy.

Variable	Treated No (%)	Cure No (%)	Failure No (%)	Univar ate OR(95%CI)	P-value	Multivariate OR(95%CI)	P-value
**Sex**							
Male	541 (48.5)	481 (88.9)	60 (11.1)	1.668(1.098–2.536)	0.017	1.708 (1.108–2.632)	0.015
Female	575 (51.5)	535 (93)	40 (7)	1		1	
**Age**							
>25	621 (55.6)	551 (88.7)	70 (11.3)	1.969(1.262–3.073)	0.003	1.865 (1.182–2.943)	0.007
≥25	495 (44.4)	465 (93.9)	30 (6.1)	1		1	
**Nationality**							
Iranian	1011 (90.6)	920 (91)	91 (9)	1.055(0.515–2.16)	0.883		
Afghani	105 (9.4)	96 (91.4)	9 (8.6)	1			
**Location**							
Face	246 (22)	219 (89)	27 (11)	1.346(0.845–2.145)	0.211		
Others	870 (78)	797 (91.6)	73 (8.4)	1			
**Number of lesions**							
1	747 (66.9)	683 (91.4)	64 (8.6)	1			
≥2	369 (33.1)	333 (90.2)	36 (9.8)	1.154(0.751–1.771)	0.513		
**Size of lesion (mm)**							
≤10	704 (63.1)	637 (90.5)	67 (9.5)	1.208(0.781–1.868)	0.395		
>10	412 (36.9)	379 (92)	33 (8)	1			
**Dressing of the lesion**							
Yes	893 (80)	811 (90.8)	80 (9.2)	1.15(0.676–1.962)	0.604		
No	223 (20)	205 (91.9)	18 (8.1)	1			
**Treatment regimen**							
Complete treatment adherence	1086 (97.3)	993 (91.4)	93 (8.6)	1		1	
Poor treatment adherence	30 (2.7)	23 (76.7)	7 (23.3)	3.25(1.358–7.77)	0.008	3.016 (1.22–7.447)	0.017
**Duration of lesion** **(month)**							
≤4	672 (60.2)	633 (94.2)	39 (5.8)	1		1	
>4	444 (39.8)	383 (86.3)	61 (13.7)	2.585(1.696–3.94)	≤ 0.001	2.679(1.745–4.112)	≤ 0.001

### Intramuscular administration

Among the ACL patients (n = 275), multivariate method validated one identical risk factor consisting of duration > 4 months of the lesion (OR = 2.063, CI = 1.019–4.175, p = 0.44) was significantly associated with treatment failure (Table 4).

**Table 4:** Associated-risk determinants for anthroponotic cutaneous leishmaniasis patients treated by intramuscular meglumine antimoniate alone.

Variable	Treated No (%)	Cure No (%)	Failure No (%)	Univar ate OR(95%CI)	P-value	Multivariate OR(95%CI)	P-value
**Sex**							
Male	160(58.2)	128(80)	32(20)	1.263(0.675–2.363)	0.465		
Female	115(41.8)	96(83.5)	19(16.5)	1			
**Age**							
>25	145(52.7)	119(82.1)	26(17.9)	1.09(0.593–2.003)	0.782		
≥25	130(47.3)	105(80.8)	25(19.2)	1			
**Nationality**							
Iranian	234(85.1)	186(79.5)	48(20.5)	3.269(0.967–11.044)	0.057		
Afghani	41(14.9)	38(92.7)	3(7.3)	1			
**Location**							
Face	111(40.4)	86(77.5)	25(22.5)	1.543(0.837–2.844)	0.165		
Others	164(59.6)	138(84.1)	26(15.9)	1			
**Number of lesions**							
1	143(52)	118(82.5)	25(17.5)	1			
≥2	132(48)	106(80.3)	26(19.7)	1.158(0.630–2.127)	0.637		
**Size of lesion (mm)**							
≤10	103(37.5)	81(78.6)	22(21.4)	1.339(0.72–2.484)	0.35		
>10	172(62.5)	143(83.1)	29(16.9)	1			
**Dressing of the lesion**							
Yes	184(66.9)	73(80.2)	18(19.8)	1.128(0.596–2.137)	0.711		
No	91(33.1)	151(82.1)	33(17.9)	1			
**Treatment regimen**							
Complete treatment adherence	205(74.5)	169(82.4)	36(17.6)	1			
Poor treatment adherence	70(25.5)	55(78.6)	15(21.4)	1.28(0.652–2.514)	0.473		
**Duration of lesion** **(month)**							
≤4	98(35.6)	86(87.8)	12(12.2)	1		1	
>4	177(64.4)	138(78)	39(22)	2.025(1.005–4.082)	0.048	2.063(1.019–4.175)	0.044

### Survival estimates

**Fig 3:**
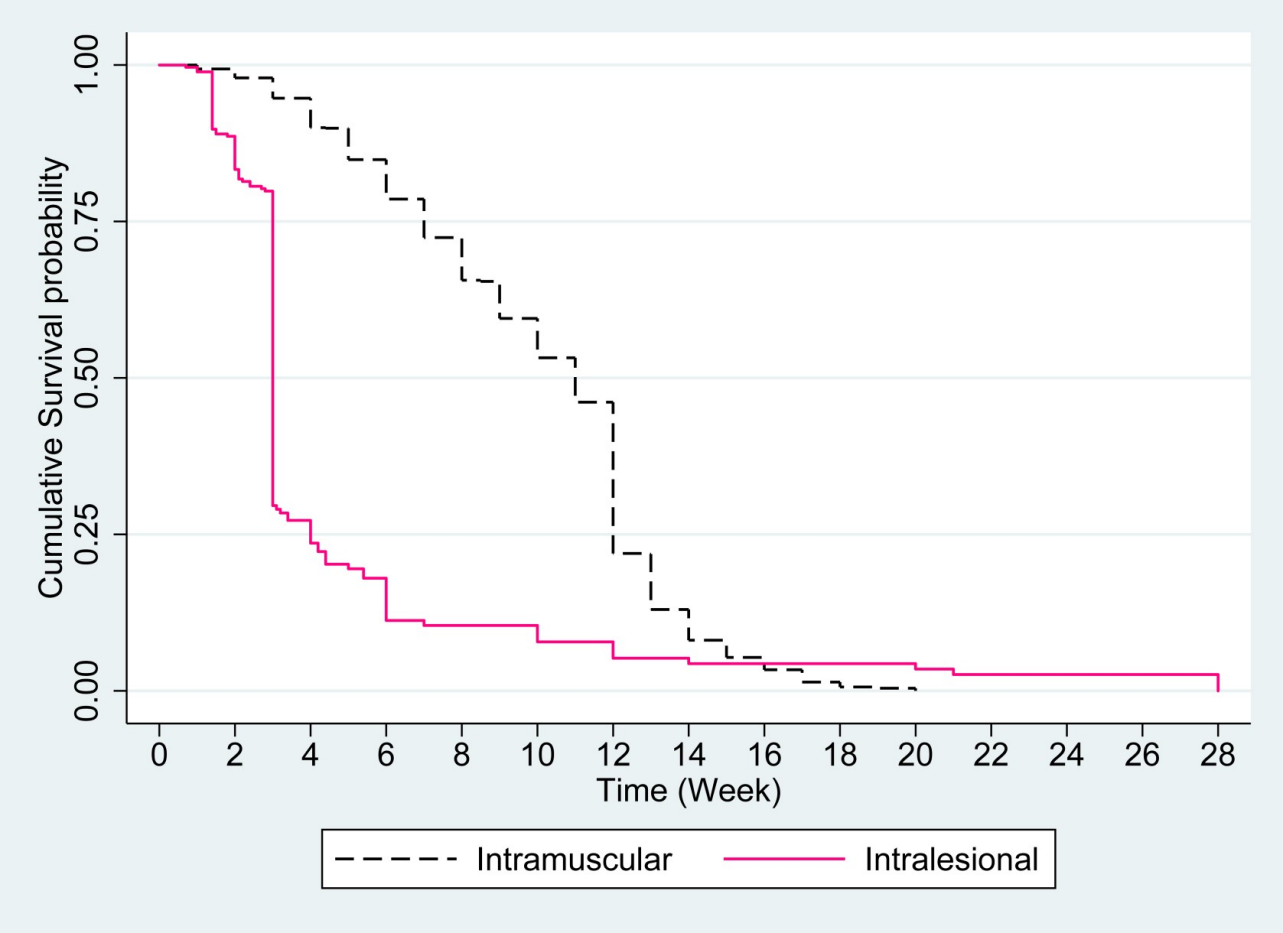
Comparison of survival rate of intramuscular and intralesional prescription of meglumine antimoniate over time.

Kaplan-Meier graphical checks revealed that intralesional administration of meglumine antimoniate included cure of the lesion(s) more rapidly compared to intramuscular administration. Kaplan-Meier graph showed around 75% of those with intralesional administration cured around four weeks after starting treatment, however this time and percentage for intramuscular administration was around 12 weeks after initiating treatment (p ≤0.001) (Fig 3).

### Molecular finding

**Fig 4:**
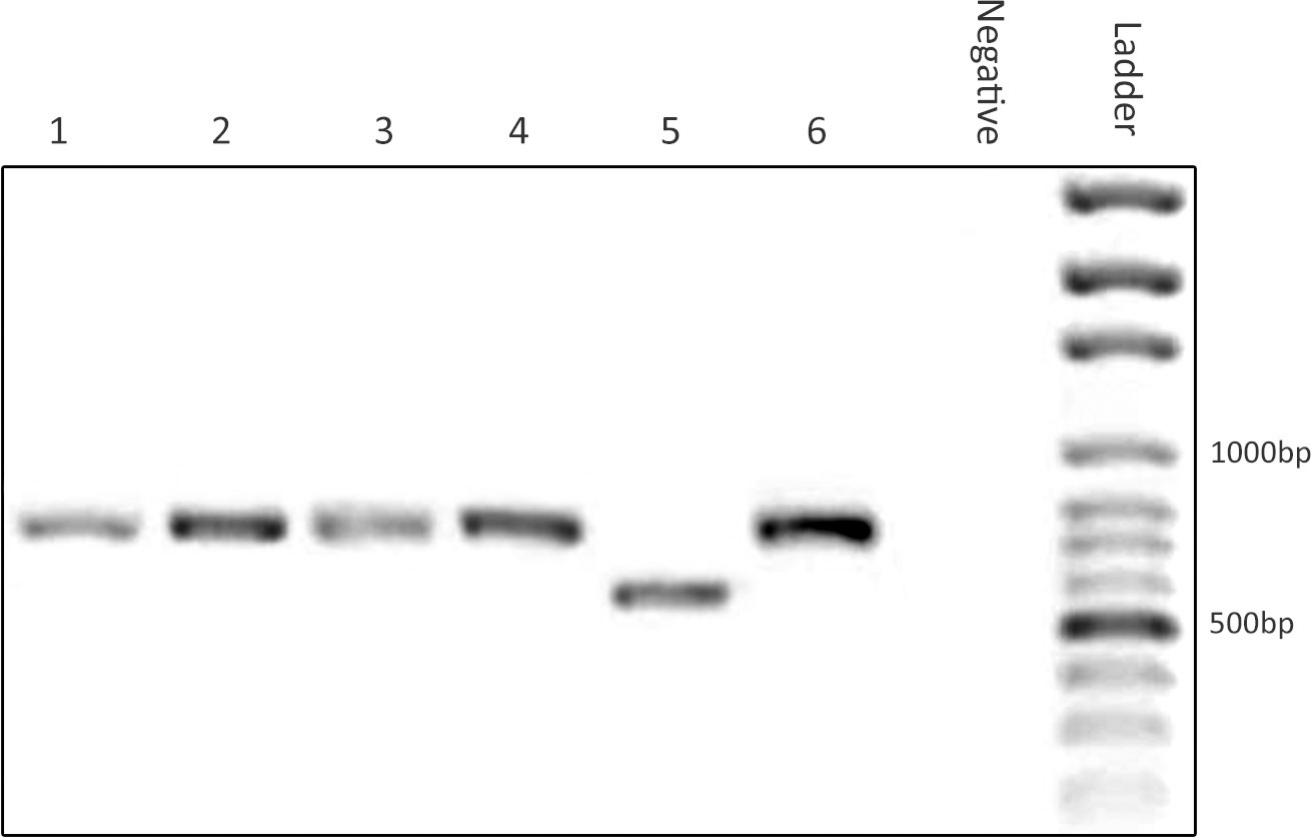
Identification of *Leishmania* species by nested PCR to amplify a variable minicircle region of kinetoplat DNA (kDNA) prepared on gel electrophoresis products. Ladder: DNA size marker,100 bp; Negative: Negative control (distilled water); 6. Positive control (*Leishmania tropica*, 750 bp); 5. Positive control (*Leishmania major*, 560 bp); Representative isolates (1–5) obtained from the patients with anthroponotic cutaneous leishmaniasis, southeast Iran.

The molecular results of nested PCR exhibited that all 207 randomly selected isolates were confirmed to be *L*. *tropica*, the causative agent of ACL in the Old World (Fig 4).

## Discussion

In the present study, the current MA therapy demonstrated an overall cure rate of 89% (n = 1,240/1,391cases) using a combination of IL plus cryotherapy and IM. However, the risk of failure was significantly more common among IM MA treatment (18.6%) in comparison with IL route (9%) by only univariate analysis, although the rate of relapse was similar in both routes of therapy. Moreover, the speed of recovery was higher in IL administration than the IM route. Cryotherapy alone is effective to treat CL lesions. This additional therapy could contribute to such a difference [19,20]. The mode of action by which cryotherapy destroys cells is compound, it consist of a rapid freeze, which in turn produces extremely damaging intracellular ice creation and ultimately cellular destruction [19].

The failure rate was comparable for ACL-affected patients following the completion of one course of therapy in Central Iran [21] and Peru [22]. On the other hand the treatment failure was different from the patients from Pakistan [23]. These variable ranges of treatment failures are attributed to multiple factors such as *Leishmania* species, different dosages, treatment schedules and evaluation time [24].

It should be noted that cases of re-infections may be confused with relapse in ACL endemic areas. However, recent evidence substantiates the general notion that recurrence is certainly the cause of leishmaniasis recidivans (lupoid leishmaniasis; LR) which is seen in ACL patients following successful treatment [25]. Leishmaniasis recidivans is usually a sequel of ACL infection caused by *L*. *tropica* and may last for many years and rarely respond to conventional treatment. Although it is difficult to show amastigotes in the lesion by direct smear microscopy, Leishman bodies remain in the lesion for several sessions of transmission cycles and therefore serve as a source of reservoir parasite to infect female sand flies [14]. An incidence rate of 18.7% among ACL cases has already been recorded in endemic foci in school age children [13] and 4.7% in all age groups and predominantly in male individuals [26].

A possible explanation for males being at higher risk of treatment failure than females might be attributed to the immunological status of the host. There are fundamental differences in innate and adaptive immune responses of both sexes in the pathogenesis of infectious diseases. Females elicit a more robust and efficient response to a wide range of microbial and parasitic infections [27]. It is clear that an efficient cell-mediated responses is essential for rapid parasite clearance and for maximal efficacy of pentavalent antimonial compounds in the treatment of leishmaniasis [28]. Moreover, such sex-based immunological divergence contributes to variations in the incidence of infectious diseases. The precise association between gender and the risk of CL is complex and needs to be investigated further.

The exact reasons for face particularly in males being the main risk factor for treatment failure are not well understood. Based on our findings most of the lupoid leishmaniasis manifestation (LR) occurred on the face of male patients. This clinical presentation is a common form of ACL caused by *L*. *tropica* in the Old World [13,29]. The majority of the LR lesions are highly resistant to pentavalent antimonials [30,31]. Several precipitating factors have been implicated as the cause of LR. Male patients with ACL often do not complete the standard course of therapy with MA, largely due to partial treatment adherence. Such low adherence to treatment might be the primary reason for the development of failure. Other contributing factors could be involved in the generation of such resistant features as the result of specific types of immune response, the causative species and even due to particular characteristics of genetic variants. Previous investigations reported two *L*. *tropica* genotypes (Mon-39 and Mon-55) from this area [29].

Among determinants of treatment outcome, multiple lesions possibly play an important role in the failure of the disease compared with single lesion. Various studies have already documented that the failure rate considerably increases with each additional skin lesion. Hence, multiple lesions could be a reflex of high parasite burden and, in turn, impair *Leishmania* clearance [32]. There may be multiple lesions notably when the patient has faced a nest of sand flies as the consequence of multiple female sand flies bites [33,34]. In this instance presumably different genotypes with diverse susceptibility levels could be introduced to the susceptible host. Such genotype differences within the same species might behave differently to chemotherapy, as a number of intraspecific genetic variants have already been documented for *L*. *tropica* from the same area [7,35,36]. Also failure caused by patients bearing multiple lesions could be a consequence of a decreased capability of the immune system against the *Leishmania* species [32].

Adherence to treatment has a profound influence in the consequence of chemotherapy; however, poor compliance declines optimum outcome. The present finding represents that adherence to both treatment modalities (IL plus cryotherapy and IM alone) was a major challenge for patients and healthcare providers, as a significant number of cases did not follow advice from the physicians and primary health care providers. This problem remains a limiting factor in the achievement of the therapeutic cure. Various reports indicate that across a wide variety of clinical settings and treatment recommendations, an important number of patients do not comply with the treatment regimen and schedule [37]. Poor adherence to the treatment occurs for a wide variety of reasons including doubt about the expected benefits, efficacy of treatment, unpleasant side-effects, work constraints or economic situation, traveling away from home, feeling sick or depressed and simple forgetfulness [38]. In the present study a considerable number of patients showed difficulty in adhering to their recommended therapeutic regimens. This fact represents the mismanagement of MA in the endemic areas of the present study as major contributor to the development of failure. Treatment failure should be monitored in order to maintain the life time of existing antileishmanial drugs, their delivery and clinical response [15].

It is worth mentioning that cases with shorter durations of lesions (≤ 4 months) showed a lower risk of failure. Importantly, lesions caused by *L*. *tropica* have the tendency to undertake chronic course and in some occasions, the lesion develop to LR lesion, which is extremely resistant to standard treatments [13,14,31]. In addition, management of the disease within few weeks of onset is not beneficial due to inadequate effector response, as this significantly increases the failure rate [22,39]. It is found that early treatment within 5 weeks of the infection onset significantly enhances the failure rate to 47% [22]. A previous investigation by Machado and colleagues reported a similar treatment failure (46%) [40].

In general, the problem of treatment failure has been rather neglected in the mainstream delivery of primary healthcare services. A robust commitment to a multidisciplinary approach is necessary in order to make advancement in this area. This will require coordinated action from health professionals, researchers, health providers and policy–makers. Treatment outcome is a multidimensional phenomenon [41] determined by the interplay of compound factors. No single intervention has been shown to be effective for all the patients, conditions and settings. Consequently, physicians and healthcare surveillance systems need to develop means of accurately assessing not only adherence, but also those determinants which contribute to treatment failure [38].

To the best of our knowledge, this cohort study represents the first and largest observation to assess treatment risk determinants in a large scale number of patients with ACL in Iran. The main strength of the present study is CL treatment center with a robust registry system and adequate infrastructure, which manages the patients through a team of well-trained, experienced physicians and health personnel and well equipped diagnostic laboratory facilities, supported by the Leishmaniasis Research Center which is affiliated with the Kerman University of Medical Sciences. On the other hand, the present study had a number of limitations. First, although, CL is a part of primary health care (PHC) surveillance system, one limitation was the lack of active case-detection approaches in a systematic manner to follow-up the patients through house–to–house visits due to the vast and remote areas of coverage. An active case-detection strategy could assist in determining the actual burden of ACL in the area. This would help in planning future control strategies. However, the patients had been advised to report any suspected skin lesion resembling CL at the earliest time, following the schedule. Second, another limitation was the low number of patients who received IM therapy in this study. Due to the presence of solitary skin lesions in ACL (average; 1.5); inevitably, the majority of cases fell in the IL route of administration along with cryotherapy. Third, a considerable number of eligible patients who were primarily screened and entered in the cohort did not meet the inclusion criteria and therefore were excluded (n = 501/1,892, 26.5%) during the course of treatment and follow up examinations. Nevertheless, the number of patients were considerably high and they came from different areas within the County, as one of the main focus of ACL in the country and this increases the general validity of the study. Four, finally the last caveat has to be mentioned; we randomly confirmed 207 samples that were obtained from the patients (14.9% out of 1,391).

Finally, the advance of more effective drugs for the treatment of ACL is extremely important because, at present, chemotherapy is the only measure to control the disease for reducing the reservoir of infection in endemic areas. Combination therapy is now being recommended for many infectious diseases, including ACL. This modality offers the potential of preventing drug resistance, because organisms resistant to one of the drugs may be susceptible to the other drug; and also the potential to diminish drug therapy duration and thus side effects [42].

In conclusion, treatment failure is a complex and multifactorial phenomenon; therefore, a comprehensive intervention and coordinated action by the health authorities and policy-makers are crucial to make sure that patients strictly follow medical instructions. Early detection and effective therapy < 4 months following the onset of the lesion is critical for successful treatment of the patients. Since primary reasons for the major determinants of treatment failure and relapse are not yet well understood, additional efforts is more likely advisable to focus on ACL education and preventive measures which might protect high risk people and reduce man-vector exposure. The present findings also highlight an urgent need for new effective alternative drugs against ACL caused by *L*. *tropica* for planning future therapeutic strategies.

## Supporting information

S1 Checklist STROBE checklist. (PDF)

S1 Table SD, mean and numbers based on demographic and clinical characteristics. (DOCX)
