# Supplementary material for: Associated-risk determinants for anthroponotic cutaneous leishmaniasis treated with meglumine antimoniate: A cohort study in Iran
Source: PLoS Negl Trop Dis. 2019 Jun 12;13(6):e0007423. doi: 10.1371/journal.pntd.0007423 (PMC6590833; doi:10.1371/journal.pntd.0007423)
Supplement: S1 Checklist — (PDF) [file pntd.0007423.s001.pdf]

STROBE Statement—checklist of items that should be included in reports of observational studies

	Item No		Recommendation
**Title and abstract**	1	(*a*) Indicate the study's design with a commonly used term in the title or the abstract	(*a*) Done "Title", Line 2 and "Abstract", "Methodology", First Line.
		(*b*) Provide in the abstract an informative and balanced summary of what was done and what was found	(*b*) Done "Abstract", "Methodology", Whole paragraph.
		**Introduction**	
Background/rationale	2	Explain the scientific background and rationale for the investigation being reported	Done "Introduction", Whole paragraphs.
Objectives	3	State specific objectives, including any prespecified hypotheses	Done "Introduction", Last paragraph.
		**Methods**	
Study design	4	Present key elements of study design early in the paper	Done "Methods", "Design and patient recruitments", Whole paragraphs.
Setting	5	Describe the setting, locations, and relevant dates, including periods of recruitment, exposure, follow-up, and data collection	Done "Methods", From "Design and patient recruitments" to "Statistical analysis".
Participants	6	(*a*) *Cohort study*—Give the eligibility criteria, and the sources and methods of selection of participants. Describe methods of follow-up<br>*Case-control study*—Give the eligibility criteria, and the sources and methods of case ascertainment and control selection. Give the rationale for the choice of cases and controls<br>*Cross-sectional study*—Give the eligibility criteria, and the sources and methods of selection of participants	(*a*) Done "Methods", "Design and patient recruitments", Whole paragraphs and Fig 1.
		(*b*) *Cohort study*—For matched studies, give matching criteria and number of exposed and unexposed<br>*Case-control study*—For matched studies, give matching criteria and the number of controls per case	(*b*) Done "Methods", "Design and patient recruitments" Whole paragraphs and Fig 1.
Variables	7	Clearly define all outcomes, exposures, predictors, potential confounders, and effect modifiers. Give diagnostic criteria, if applicable	Done "Methods", From "Design and patient recruitments" to "Statistical analysis".

Data sources/ measurement	8*	For each variable of interest, give sources of data and details of methods of assessment (measurement). Describe comparability of assessment methods if there is more than one group	Done “Methods", From “Design and patient recruitments” to “Statistical analysis” and Fig 1.
Bias	9	Describe any efforts to address potential sources of bias	Done "Methods", "Design and patient recruitments", Whole paragraphs and Fig 1.
Study size	10	Explain how the study size was arrived at	Done "Methods", "Design and patient recruitments", Whole paragraphs and Fig 1.
Quantitative variables	11	Explain how quantitative variables were handled in the analyses. If applicable, describe which groupings were chosen and why	Done “Methods", From “Design and patient recruitments” to “Statistical analysis” and Tables.
Statistical methods	12	(*a*) Describe all statistical methods, including those used to control for confounding	(*a*) Done “Methods", “Statistical analysis”, Whole paragraphs.
		(*b*) Describe any methods used to examine subgroups and interactions	(*b*) Done “Methods", “Statistical analysis”, Whole paragraphs.
		(*c*) Explain how missing data were addressed	(*c*) Done “Methods", "Design and patient recruitments” Whole paragraphs and Fig 1.
		d) *Cohort study*—If applicable, explain how loss to follow-up was addressed<br>*Case-control study*—If applicable, explain how matching of cases and controls was addressed<br>*Cross-sectional study*—If applicable, describe analytical methods taking account of sampling strategy	(*d*) Done “Methods", "Design and patient recruitments”, Whole paragraphs and Fig 1.
		(*e*) Describe any sensitivity analyses	(*e*) No.

Continued on next page

		Results	
Participants	13*	(a) Report numbers of individuals at each stage of study—eg numbers potentially eligible, examined for eligibility, confirmed eligible, included in the study, completing follow-up, and analysed	(a) Done "Results", First paragraph and Fig 1.
		(b) Give reasons for non-participation at each stage	(b) Done "Results", First paragraph and Fig 1.
		(c) Consider use of a flow diagram	(c) Done "Results", Fig 1.
Descriptive data	14*	(a) Give characteristics of study participants (eg demographic, clinical, social) and information on exposures and potential confounders	(a) Done "Results", "Demographical characteristics" to "Molecular finding", Whole paragraphs, Fig 2 and Tables 1-4.
		(b) Indicate number of participants with missing data for each variable of interest	(b) Done "Results", Fig 1.
		(c) *Cohort study*—Summarise follow-up time (eg, average and total amount)	(c) Done "Results", First paragraph.
Outcome data	15*	*Cohort study*—Report numbers of outcome events or summary measures over time	Done "Results", Tables 2-4 and Fig 3.
		Case-control study—Report numbers in each exposure category, or summary measures of exposure	
		Cross-sectional study—Report numbers of outcome events or summary measures	
Main results	16	(*a*) Give unadjusted estimates and, if applicable, confounder-adjusted estimates and their precision (eg, 95% confidence interval). Make clear which confounders were adjusted for and why they were included	(*a*) Done "Results", "Demographical characteristics" (whole paragraph), "Clinical status" (whole paragraphs), "Treatment Outcome" (whole paragraph), "Risk determinants" (whole paragraph), "Treatment with combined intralesional and intramuscular MA" (whole paragraphs).
		(*b*) Report category boundaries when continuous variables were categorized	(*b*) Done "Results", Tables.
		(*c*) If relevant, consider translating estimates of relative risk into absolute risk for a meaningful time period	(*c*) No.
Other analyses	17	Report other analyses done—eg analyses of subgroups and interactions, and sensitivity analyses	Done "Results", "Treatment with combined intralesional and intramuscular MA", Second paragraph.
		**Discussion**	
Key results	18	Summarise key results with reference to study objectives	Done "Discussion", First paragraph.
Limitations	19	Discuss limitations of the study, taking	Done "Discussion", Last two paragraphs

		into account sources of potential bias or imprecision. Discuss both direction and magnitude of any potential bias	before “In conclusion”.
Interpretation	20	Give a cautious overall interpretation of results considering objectives, limitations, multiplicity of analyses, results from similar studies, and other relevant evidence	Done “Discussion", Whole paragraphs.
Generalisability	21	Discuss the generalisability (external validity) of the study results	Done “Discussion", Whole paragraphs.
		**Other information**	
Funding	22	Give the source of funding and the role of the funders for the present study and, if applicable, for the original study on which the present article is based	The authors received no specific funding for this study.

*Give information separately for cases and controls in case-control studies and, if applicable, for exposed and unexposed groups in cohort and cross-sectional studies.

Note: An Explanation and Elaboration article discusses each checklist item and gives methodological background and published examples of transparent reporting. The STROBE checklist is best used in conjunction with this article (freely available on the Web sites of PLoS Medicine at http://www.plosmedicine.org/, Annals of Internal Medicine at http://www.annals.org/, and Epidemiology at http://www.epidem.com/). Information on the STROBE Initiative is available at www.strobe-statement.org.
