# Supplementary material for: Associated-risk determinants for anthroponotic cutaneous leishmaniasis treated with meglumine antimoniate: A cohort study in Iran
Source: PLoS Negl Trop Dis. 2019 Jun 12;13(6):e0007423. doi: 10.1371/journal.pntd.0007423 (PMC6590833; doi:10.1371/journal.pntd.0007423)
Supplement: S1 Table — (DOCX) [file pntd.0007423.s002.docx]

**S1 Table.**

**Mean and SD**

|  | | | **Number of lesion** | | | **Size of lesion( mm )** | | **Age** |  |
| --- | --- | --- | --- | --- | --- | --- | --- | --- | --- |
| **sex** | | |  | | |  | |  |  |
| **male** | | Mean | 1.6690 | | | 19.3773 | | 22.4069 |  |
|  |  | N | 701 | | | 701 | | 701 |  |
|  |  | Std. Deviation | 1.25312 | | | 18.43054 | | 17.61412 |  |
| **female** | | Mean | 1.7797 | | | 16.2551 | | 28.7413 |  |
|  |  | N | 690 | | | 690 | | 690 |  |
|  |  | Std. Deviation | 1.60121 | | | 13.74583 | | 20.49274 |  |
| **Total** | | Mean | 1.7239 | | | 17.8285 | | 25.5491 |  |
|  |  | N | 1391 | | | 1391 | | 1391 |  |
|  |  | Std. Deviation | 1.43692 | | | 16.34507 | | 19.35060 |  |
| **nationality** | | |  | | |  | |  |  |
| **Iranian** | | Mean | 1.6964 | | | 17.7305 | | 26.5693 |  |
|  |  | N | 1245 | | | 1245 | | 1245 |  |
|  |  | Std. Deviation | 1.38354 | | | 16.41322 | | 19.55124 |  |
| **Afghani** | | Mean | 1.9589 | | | 18.6644 | | 16.8493 |  |
|  |  | N | 146 | | | 146 | | 146 |  |
|  |  | Std. Deviation | 1.81897 | | | 15.78182 | | 14.99740 |  |
| **Total** | | Mean | 1.7239 | | | 17.8285 | | 25.5491 |  |
|  |  | N | 1391 | | | 1391 | | 1391 |  |
|  |  | Std. Deviation | 1.43692 | | | 16.34507 | | 19.35060 |  |
| **location (face and Other)** | | |  | | |  | |  |  |
| **face** | | Mean | 1.3725 | | | 16.7703 | | 20.8371 |  |
|  |  | N | 357 | | | 357 | | 357 |  |
|  |  | Std. Deviation | .78870 | | | 13.99711 | | 21.60333 |  |
| **Other** | | Mean | 1.8453 | | | 18.1939 | | 27.1759 |  |
|  |  | N | 1034 | | | 1034 | | 1034 |  |
|  |  | Std. Deviation | 1.58320 | | | 17.07195 | | 18.23983 |  |
| **Total** | | Mean | 1.7239 | | | 17.8285 | | 25.5491 |  |
|  |  | N | 1391 | | | 1391 | | 1391 |  |
|  |  | Std. Deviation | 1.43692 | | | 16.34507 | | 19.35060 |  |
| **Number of lesion 1 and 2<** | | |  | | |  | |  |  |
| **1** | | Mean | - | | | 18.2770 | | 24.0451 |  |
|  |  | N | - | | | 890 | | 890 |  |
|  |  | Std. Deviation | - | | | 17.49601 | | 19.03545 |  |
| **≥ 2** | | Mean | - | | | 17.0319 | | 28.2208 |  |
|  |  | N | - | | | 501 | | 501 |  |
|  |  | Std. Deviation | - | | | 14.05173 | | 19.63482 |  |
| **Total** | | Mean | - | | | 17.8285 | | 25.5491 |  |
|  |  | N | - | | | 1391 | | 1391 |  |
|  |  | Std. Deviation | - | | | 16.34507 | | 19.35060 |  |
| **Size of lesion (mm)** | | |  | | |  | |  |  |
| **≤ 10** | | Mean | 1.7522 | | | - | | 24.4021 |  |
|  |  | N | 807 | | | - | | 807 |  |
|  |  | Std. Deviation | 1.46778 | | | - | | 19.12626 |  |
| **> 10** | | Mean | 1.6849 | | | - | | 27.1341 |  |
|  |  | N | 584 | | | - | | 584 |  |
|  |  | Std. Deviation | 1.39346 | | | - | | 19.56232 |  |
| **Total** | | Mean | 1.7239 | | | - | | 25.5491 |  |
|  |  | N | 1391 | | | - | | 1391 |  |
|  |  | Std. Deviation | 1.43692 | | | - | | 19.35060 |  |
| **Age** | | | |  |  | |  | | |
| **< 25** | Mean | | | 1.6044 | 16.9726 | | - | | |
|  | N | | | 766 | 766 | | - | | |
|  | Std. Deviation | | | 1.25617 | 16.24431 | | - | | |
| **≥ 25** | Mean | | | 1.8704 | 18.8776 | | - | | |
|  | N | | | 625 | 625 | | - | | |
|  | Std. Deviation | | | 1.62043 | 16.41991 | | - | | |
| **Total** | Mean | | | 1.7239 | 17.8285 | | - | | |
|  | N | | | 1391 | 1391 | | - | | |
|  | Std. Deviation | | | 1.43692 | 16.34507 | | - | | |
